# Genome-Wide *In Silico* Identification and Comparative Analysis of *Dof* Gene Family in *Brassica napus*

**DOI:** 10.3390/plants10040709

**Published:** 2021-04-07

**Authors:** Neeta Lohani, Saeid Babaei, Mohan B. Singh, Prem L. Bhalla

**Affiliations:** Plant Molecular Biology and Biotechnology Laboratory, Faculty of Veterinary and Agricultural Sciences, The University of Melbourne, Parkville, Melbourne, VIC 3010, Australia; nlohani@student.unimelb.edu.au (N.L.); sbabaeighara@student.unimelb.edu.au (S.B.); mohan@unimelb.edu.au (M.B.S.)

**Keywords:** *Dof*, *Brassica napus*, canola, transcription factor, polyploidy, abiotic stress

## Abstract

DNA binding with one finger (DOF) proteins are plant-specific transcription factors that play roles in diverse plant functions. However, little is known about the DOF protein repertoire of the allopolyploid crop, *Brassica napus*. This *in silico* study identified 117 *Brassica napus Dof* genes (*BnaDofs*) and classified them into nine groups (A, B1, B2, C1, C2.1, C2.2, C3, D1, and D2), based on phylogenetic analysis. Most members belonging to a particular group displayed conserved gene structural organisation and protein motif distribution. Evolutionary analysis exemplified that the divergence of the *Brassica* genus from Arabidopsis, the whole-genome triplication event, and the hybridisation of *Brassica oleracea* and *Brassica rapa* to form *B. napus*, followed by gene loss and rearrangements, led to the expansion and divergence of the *Dof* transcription factor (TF) gene family in *B. napus*. So far, this is the largest number of *Dof* genes reported in a single eudicot species. Functional annotation of BnaDof proteins, cis-element analysis of their promoters, and transcriptomic analysis suggested potential roles in organ development, the transition from the vegetative to the reproductive stage, light responsiveness, phytohormone responsiveness, as well as potential regulatory roles in abiotic stress. Overall, our results provide a comprehensive understanding of the molecular structure, evolution, and possible functional roles of *Dof* genes in plant development and abiotic stress response.

## 1. Introduction

*Brassica napus,* the second-largest economically important oilseed crop, is used as edible oil and livestock forage, and in the pharmaceuticals, cosmetics, and biofuel industries [1]. The yield of *B. napus* is constrained by harsh environmental conditions such as drought, extreme temperature, and salinity [2]. Dissecting the evolution and function of diverged plant-specific transcription factor (TF) families such as DNA binding with one finger (DOF) is required for gaining fundamental knowledge about the mechanisms underlying stress responses in *B. napus* and for developing stress-tolerant varieties for climate-smart agriculture.

The DOF TFs are plant-specific transcription factors, first identified in maize in 1995, and were shown to play an essential role in regulating carbon metabolism-related and light-regulated genes [3,4,5]. Subsequently, a diverse number of DOF TFs have been identified in several plants, including 36, 30, and 96 in Arabidopsis, rice, and wheat, respectively [6,7,8]. In Arabidopsis, the first protein–protein interaction of DOF domain protein with bZIP protein associated with the stress response was reported, indicating the potential role of DOF TFs in complex regulatory networks [9].

DOF TFs are typically composed of 200–400 amino acid residues with a variable C-terminal region and are mainly characterised by a highly conserved DNA-binding domain, i.e., the DOF domain, located towards the N-terminal of the protein [10,11]. The DOF domain consists of only one Cys2/Cys2 zinc finger structure, which includes about 52 amino acid residues, that specifically recognises and binds a cis-regulatory element (5′-T/AAAAG-3′) in the gene promoters [12]. Another specific feature of DOF TFs is a bipartite nuclear localisation signal (NLS) comprised of two short basic flanking regions (B1 and B2) with a 17 amino acid spacer. The bipartite NLS is highly conserved and plays a vital role in directing DOF TFs to the cell nucleus [13].

Phylogenetic studies suggest a common ancestor (conserved as a single copy in *Chlamydomonas*) of *Dof* genes, which through numerous rounds of gene duplication drove the structural and functional diversification of the *Dof* gene family [12]. This evolutionary diversification may be related to acquiring new specialised functions needed to adjust and adapt to diverse and complex plant growth conditions. *Brassica napus*, an amphidiploid (AACC, 2n = 4x = 38), originated from a natural crossing between two ancestral diploid parents, *Brassica oleracea* (CC, 2n = 2x = 18) and *Brassica rapa* (AA, 2n = 2x = 20), approximately 7500 years ago [14]. *Brassica* species belonging to the *Brassica* genus and Brassicaceae family offer a valuable model for studying polyploid genome evolution, mechanisms associated with gene duplications, loss of duplicated genes and gene neo- and sub- functionalisation [15,16]. The availability of *B. napus*, *B. rapa*, and *B. oleracea* genome sequences has provided an exceptional opportunity to identify and characterise key genes from a genome-wide perspective [14,16,17].

This study aims to provide a complete description of the *Dof* gene family in *B. napus* by performing a genome-wide in silico identification, characterisation, and evolutionary and functional analysis of the *Dof* gene family. We report identifying one hundred seventeen genes as members of the *Dof* transcription factor family from *B. napus*, belonging to nine groups. We also performed a detailed analysis of the *Dof* genes in terms of physical properties of proteins, chromosomal location, gene structure, motif analysis and phylogenetic relationships, and gene duplication. Orthology and synteny analysis was also carried out to explore the evolutionary history and divergence of the *Dof* TFs family in *B. napus, B. rapa, B. oleracea*, and Arabidopsis. Furthermore, functional annotation and cis-acting regulatory element analysis of *BnaDof* gene promoters and their expression profiles highlighted their potential roles in regulating distinct and diverse developmental processes and stress responses.

## 2. Results

### 2.1. Identification and Characterisation of BRASSICA Napus Dof Gene Family

**Figure 1:**
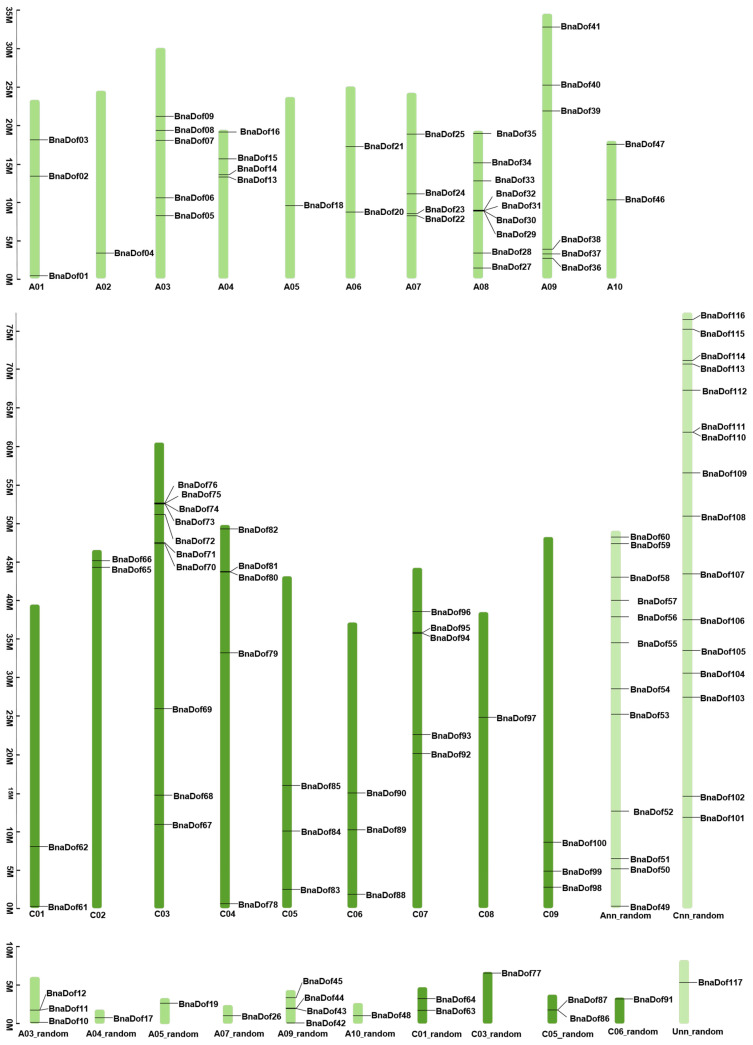
Chromosomal mapping and frequency distribution of *Brassica napus* DNA binding with one finger (DOF) transcription factors (*BnaDofs01–117).*

To identify the *Dof* TF family genes in *B. napus,* we carried out BLASTP searches using DOF-domain search model accession (Pfam: PF02701), searched the PlantTFDB4.0, and finally retrieved and analysed 156 amino acid sequences using the SMART8.0 database [18,19,20]. We identified 117 full-length *B. napus* genes as putative members of the *Dof* gene family from the *Brassica napus* reference genome (*Brassica_napus*. annotation_v5). We assigned new identifiers to the 117 *B. napus Dof* genes by using the prefix “*Bna*” for *B. napus*, followed by “*Dof*” and a number based on their chromosomal locations (Figure 1). Thus, the identified *B. napus Dof* family members were named *BnaDof01* to *BnaDof117* (Table S1). The *BnaDofs* were distributed on all 19 chromosomes, with 60 *BnaDofs* located on A genome (48 on chromosomes A01–A10), 56 situated on C genome (40 on chromosomes C01–C09), and one gene placed on an unknown chromosome (*BnaDof117*; *BnaUnng03510D*). Chromosome C03, being the largest, included the most *BnaDofs* (11 *BnaDofs*), followed by A09 with 10 *BnaDofs*. Out of 117 *BnaDof* genes, the exact chromosomal location was unknown for the 58 genes located on A03_random, A04_random, A05_random, A07_random, A09_random, A10_random, Ann_random, C01_random, C03_random, C05_random, C06_random, Cnn_random, and Unn_random chromosomes.

The physical and chemical properties of BnaDof proteins are outlined in Table S1. The size of the BnaDof protein sequences ranged from 77 (BnaDof30, BnaDof31, BnaDof75, and BnaDof76) to 453 (BnaDof33) amino acids. All BnaDofs had a marginally higher percentage of aliphatic amino acids than aromatic amino acids. The predicted isoelectric point (pI) ranged from 4.78 (BnaDof20) to 10.14 (BnaDof30, BnaDof31, BnaDof75, and BnaDof76), and the molecular weight (MW) ranged from 8.76 kD (BnaDof30, BnaDof31, BnaDof75, and BnaDof76) to 50.33 kD (BnaDof33). The Grand Average of Hydropathy (GRAVY) values of the BnaDofs reflected the hydrophilic nature of these proteins.

We also identified 62 *B. oleracea* genes as members of the *Dof* TF family (Table S2). In *B. rapa*, Ma et al. [21] reported 76 BnaDof genes. However, among *B. rapa Dof* genes, *Bra007632* contained a B3 DNA binding domain, auxin response factor domain, and Aux/IAA domain in addition to the DOF domain. Bra007632 was orthologous to *AUXIN RESPONSE FACTOR 18* (*ARF18*, *At3g61830*), and hence, we excluded it from our analysis. It is worth noting that in addition to the 117 *BnaDof* genes, one other gene (*BnaA07g02590D*) showed the presence of the DOF domain along with a Syntaxin domain. Further investigation revealed that this gene was homologous to *Bra002057*, and reported as a *B. rapa Dof* gene family member [21]. However, this gene is orthologous to Arabidopsis *SYNTAXIN OF PLANTS 21* (*SYP21*, *At5g16830*). We decided to exclude *BnaA07g02590D* and *Bra002057* from our analysis as these genes can be classified as members of the SNAP Receptor (SNARE) protein family [22]. Thus, finally, we considered 74 *Dof* genes from *B. rapa*, 62 *Dof* genes from *B. oleracea,* and 117 *Dof* genes from *B. napus*. 

### 2.2. Phylogenetic Relationships of the Dof Gene Family in B. napus

**Figure 2:**
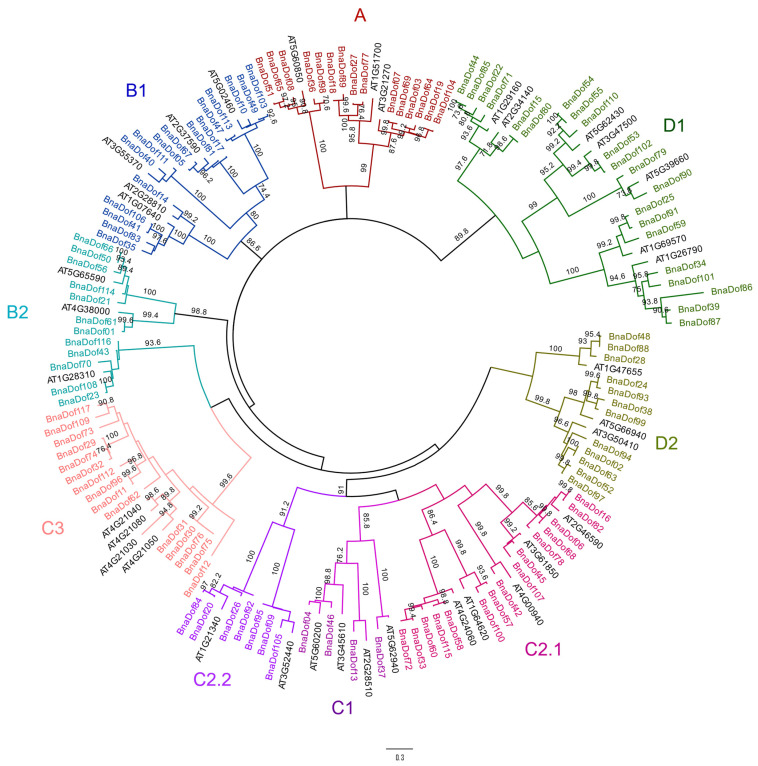
Phylogenetic tree of *B. napus* and Arabidopsis DOF proteins. The evolutionary history was inferred by using the maximum likelihood method based on the JTT (Jones-Taylor-Thornton) matrix-based method. Maximum-likelihood bootstrap values (500 replicates) above 70% are shown. The analysis involved 153 amino acid sequences. Phylogenetic topology was generated via MEGA7.

We explored the phylogenetic relationships between the *Dof* families in *B. napus* and Arabidopsis by first performing alignment of the 117 *BnaDof*s and 36 Arabidopsis *Dof*s using CLUSTALW [23]. The multiple sequence alignment was then used to construct the unrooted phylogenetic tree in MEGA7.0 [24] using the maximum likelihood method with 500 bootstrap values (Figure 2). We then classified *BnaDof*s into four major groups: A, B, C, and D and the following nine subgroups: A, B1, B2, C1, C2.1, C2.2, C3, D1, and D2, based on the phylogenetic tree. The largest group was C, with 35% of *BnaDof*s*,* followed by group D with 28% *BnaDof*s. Among the subgroups, D2 was the largest, comprising ~18% *BnaDof*s. The least number of *BnaDof*s belonged to subgroup C1. 

We further explored the phylogenetic relationship between the *Dof* gene family in *B. napus*, *B. oleracea*, *B. rapa,* and Arabidopsis. As mentioned earlier, there were 76 (74 included in our analysis) *B. rapa Dofs* and 36 Arabidopsis *Dofs,* and we identified 117 and 62 *Dof* genes in *B. napus* and *B. oleracea*, respectively [6,21]. Subsequently, a total of 289 DOF protein sequences were utilised to construct the phylogenetic tree. Based on the resulting phylogenetic tree, the *Dof* gene family can be classified into four major groups and nine subgroups, as described earlier (Figure S1). The tree illustrates the expansion and divergence of the *Dof* gene family from Arabidopsis to *B. napus*. A few genes from the two diploid progenitors were lost in *B. napus* during hybridisation, such as *Bra010136* and *Bra013490* from *B. rapa,* and *Bo3g106920 Bo01143s010* from *B. oleracea*, with no orthologs in *Brassica napus*. A few *B. rapa* genes underwent further duplications after hybridisation; for example, *Bra020880* had four orthologs in *B. napus* (*BnaDof*30, *BnaDof*31, *BnaDof*75, and *BnaDof* 76), and *Bra023888* had two orthologs in *B. napus* (*BnaDof03* and *BnaDof64*). However, the majority of *BnaDof*s were consistently inherited from their progenitors (Table S3). Based on the known chromosomal locations, we confirmed a total of 42 and 33 gene pairs which maintained their relative positions between the *B. rapa* genome and A_n_ sub-genome in the *B. napus* and *B. oleracea* genomes and C_n_ sub-genome in *B. napus*, respectively. 

### 2.3. Gene Structure and Conserved Motifs of BnaDofs

**Figure 3:**
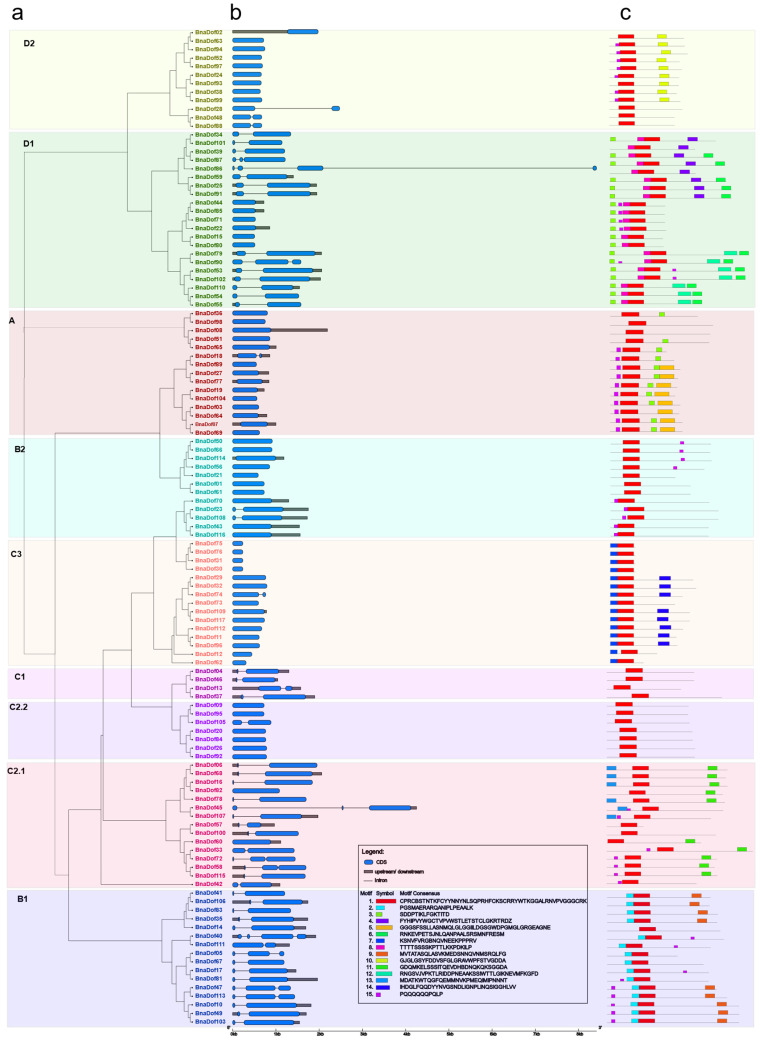
Phylogenetic relationships, gene structure, and conserved protein motifs in the *Dof* gene family members in *B. napus*. (**a**) Phylogenetic relationships of BnaDofs. The phylogenetic tree was constructed with MEGA 7.0 using the neighbour-joining (NJ) method with 1000 bootstrap replicates and the Poisson correction method. The nine *Dof* groups are displayed in different text colours and enclosed in the respective colour boxes. (**b**) Gene structure of *BnaDof* genes. Grey boxes indicate untranslated 5′- and 3′-regions; blue boxes indicate exons; and black lines indicate introns. Scale bar represents gene length. (**c**) Distribution of conserved motifs in BnaDof proteins. The sequence of each motif (1–15) displayed in different coloured boxes is provided in the legend.

To gain further understanding of the structural diversity of *BnaDof*s, we studied exon-intron organisation and identified the presence of conserved protein motifs (Figure 3). The analysis revealed 61 *BnaDof*s with no introns, whereas 46, 8, and 2 *BnaDof*s had one, two, and three introns, respectively. The majority of the genes within a given subgroup showed a similar exon-intron organisation. For example, all the C1 subgroup *BnaDof*s had one intron, and most members of subgroups D2, A, B2, C3, and C2.2 had no introns. Similarly, *BnaDof*s belonging to subgroup B1 had at least one intron. The most diverse gene structure organisation was observed in the members of subgroup D1, ranging from zero to three introns. 

Motif analysis performed using MEME showed a highly conserved motif, motif 1, representing the DOF-type domain, across all the 117 BnaDof amino acid sequences [25]. The conserved nature of motif distribution within the BnaDof protein subgroup also highlighted their phylogenetic relationships. It is worth mentioning that conserved motif distribution also existed among members of different clades within a subgroup. For example, BnaDof28, BnaDof48, and BnaDof88, which belonged to one clade in subgroup D2, showed only one out of three conserved motifs found in the other D2 subgroup members. Thus, the results suggest that gene and protein structural divergence across subgroups probably governs the functional diversity in the *Dof* subgroups. 

### 2.4. Orthologous Gene Clustering of Dof Gene Family in B. napus, B. oleracea, B. rapa, and Arabidopsis

**Figure 4:**
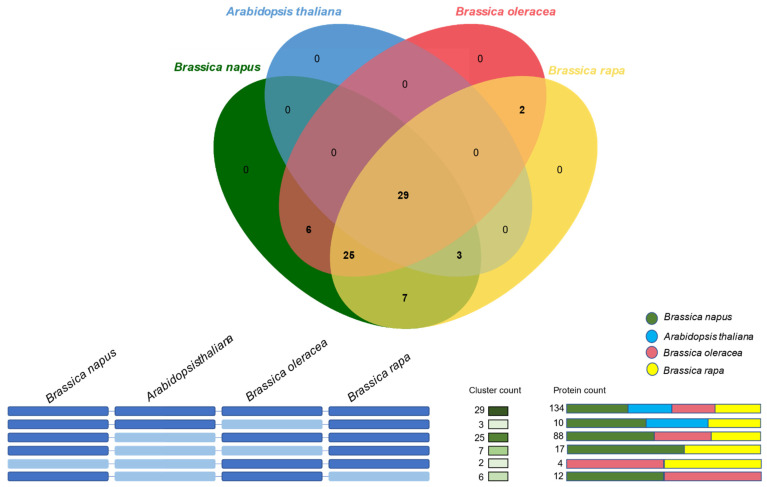
Orthologous gene clustering analysis. The orthologous gene clusters between the *Dof* gene family in *B. napus*, *B. oleracea*, *B. rapa,* and Arabidopsis were identified and visualised using the OrthoVenn2 web platform. The e-value cut-off 1e-10 was used for the analysis.

To understand the evolutionary relationships of the *Dof* gene family among the four important members of the Brassicaceae family—*B. napus*, *B. oleracea*, *B. rapa,* and Arabidopsis, we carried out an orthology analysis in OrthoVenn2 web platform [26]. The identified orthologous clusters in the four species are illustrated in Figure 4. 134 DOF proteins from all four species were clustered in 29 orthologous groups. We also identified 25 *Brassica*-specific clusters with 88 DOF proteins from the three *Brassica* species. One Arabidopsis DOF protein (AT3G45610), 17 BnaDofs, and 6 *B. rapa* DOFs did not cluster in any orthologous group and were identified as singletons. No singletons were identified in *B. oleracea*. Furthermore, there were seven clusters (17 DOFs) between *B. rapa* and *B. napus*, six clusters (12 DOFs) between *B. oleracea* and *B. napus* and two clusters between *B. rapa* and *B. oleracea*. The absence of *B. napus* genes in *B. rapa*—*B. oleracea*-specific clusters suggests that genes belonging to these clusters might have been lost during the hybridisation event. A detailed list of orthologous gene clusters and singletons is provided in Table S4a,b.

### 2.5. Evolution and Divergence of BnaDofs

The expansion of a gene family occurs because of duplication events arising at a whole-genome or small scale. We first identified duplicated gene pairs among *BnaDof*s based on the sequence similarity (Table S5a). We found 128 gene pairs with >80% sequence similarity. Gene pairs were identified as tandemly duplicated if the new gene/sequence was found adjacent (within a 100 kb window) to the duplicated genomic region. Based on these criteria, three *BnaDof* gene pairs were identified as tandemly duplicated (*BnaDof30–31, BnaDof 75–76,* and *BnaDof 86–87*). The rest of the *BnaDof* gene pairs underwent interspersed duplications. Furthermore, BLASTP- and MCScanX-based methods identified 85 segmental, 26 dispersed, 4 tandem, and 1 proximal duplication event (Table S5b) [20,27]. These results highlight that segmental duplication events played a critical role in shaping the *Dof* gene family in *B. napus*. 

Nucleotide substitutions producing an amino acid change are termed non-synonymous, and those that do not are termed synonymous. The ratio of non-synonymous to synonymous substitutions (Ka/Ks) in a protein-coding gene reflects the magnitude and direction of selection pressure acting on a protein sequence [28]. A Ka/Ks value < 1 indicates that a gene pair has experienced negative or purifying selection (acting against change), whereas Ka/Ks > 1 indicates positive or adaptive selection (driving change), and Ka/Ks = 1 indicates neutral selection [29]. Thus, we calculated the Ka/Ks ratio among the duplicated gene pairs (Table S5a). Among the 128 identified duplicated *BnaDof* gene pairs, six gene pairs (*BnaDof30* and *BnaDof31*, *BnaDof30* and *BnaDof75*, *BnaDof1*3 and *BnaDof*75, *BnaDof30* and *BnaDof 76*, *BnaDof31* and *BnaDof76*, and *BnaDof75* and *BnaDof76*) were 100% identical, and their Ka = Ks = 0. Three gene pairs (*BnaDof29* and *BnaDof74*, *BnaDof32* and *BnaDof74*, and *BnaDof72* and *BnaDof33*) underwent positive or adaptive selection (Ka/Ks > 1), and the remaining 119 gene pairs experienced negative or purifying selection (Ka/Ks < 1). Purifying selection plays a potential role in maintaining the conservation of the *Dof* genes structure during evolution. The gene pairs undergoing positive selection indicate the presence of mutations that might be advantageous for *B. napus*.

**Figure 5:**
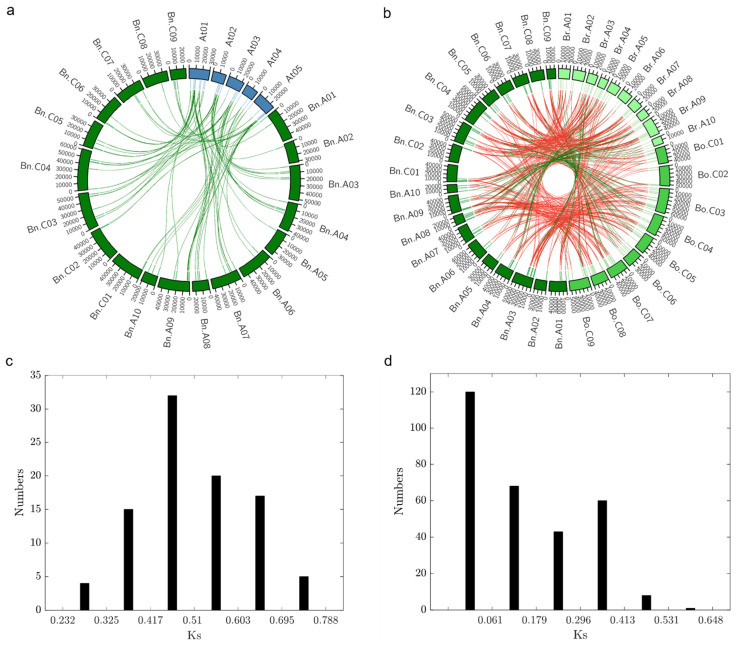
Synteny analysis (**a**) Synteny of *Dof* gene family between *Arabidopsis thaliana* and *B. napus*. Ideograms of chromosomes of *A. thaliana* (blue) and *B. napus* (dark green) are displayed in the outer circle, while in the inner circle indicates the positions of the genes in the corresponding chromosomes. Green links connect orthologous genes between the two species. The chromosome scale is in Kb. At: *Arabidopsis thaliana*; Bn: *Brassica napus*. (**b**) Synteny of DOF transcription factors within the *Brassicaceae* genus. Ideograms of chromosomes of *B. rapa* (pale green), *B. oleracea* (lime green), and *B. napus* (dark green) are displayed in the outer circle, while the inner circle indicates the positions of the genes in the corresponding chromosomes. Green links connect collinear orthologous genes, while red links connect orthologues genes that underwent chromosomal translocation events. The chromosome scale is in Kb. Bn: *Brassica napus*; Bo: *Brassica oleracea*; Br: *Brassica rapa*. (**c**) Density of Ks values of *Dof* orthologous gene pairs between *B*. *napus* and Arabidopsis. Analyses were conducted using the Nei–Gojobori model in MEGA7.0. All ambiguous positions were removed for each sequence pair. (**d**) Density of Ks values of *Dof* orthologous gene pairs between *B*. *napus*, *B. oleracea,* and *B. rapa.* Analyses were conducted using the Nei–Gojobori model in MEGA7.0. All ambiguous positions were removed for each sequence pair.

The syntenic relationships between chromosome segments of different species can provide valuable insights into the origin of the gene family members. For the synteny analysis, only the genes with known chromosomal locations were considered. We performed a syntenic analysis between *Dof* genes from *B. napus* and Arabidopsis (Figure 5a). Following our orthology analysis, we identified orthologs for 35 Arabidopsis genes in *B. napus*. In addition, we also constructed a synteny map between *Dof* genes from *B. napus*, *B. oleracea,* and *B. rapa*. 58 out of 117 genes in *B. napus* and two out of 62 genes in *B. oleracea* were discarded due to uncertain chromosomal locations (Figure 5b). Out of the remaining 59 *BnaDof*s, 98.3% of genes were placed in collinear blocks. In *B. rapa* (42/75 *Dof* genes) and in *B. oleracea* (47/60 *Dof* genes)*,* 56 and 78.3% were placed in collinear blocks, respectively. Since the same gene in *B. napus* could be in a collinear block relative to *B. rapa* but not relative to *B. oleracea*, the numbers reported for *B. rapa* and *B. oleracea* indicated how many genes were collinear with the corresponding orthologs in *B. napus*. For *B. napus*, instead, this number reported the number of collinear genes in at least one of the other species. 

Furthermore, we estimated the divergence time of the *Dof* gene family between Arabidopsis and *B. napus* by calculating the Ks values of the identified orthologous gene pairs (Table S6a). The Ks value for all the orthologous pairs ranged from 0.28 to 0.74, with an average of 0.51 (Figure 5c). Using the estimate of mutational rate, R = 1.5 × 10^−8^ synonymous substitutions per site per year [30,31], the average estimated divergence time of the Arabidopsis and *B. napus Dof* gene family was ~17 Mya. Our results agree with the reported estimated divergence time (14–24 MYA) of the Arabidopsis and *B. napus* lineage [32,33]. We also calculated the Ks values of orthologous gene pairs between the three *Brassica* species (Table S6b). The Ks value ranged from 0.0024 to 0.5896. with an average of 0.15 (Figure 5d). The average divergence time was ~5 Mya (80,000 years–19.5 Mya). The hybridisation event between *B. rapa* and *B. oleracea* took place approximately 7500–12,500 years ago, and the *Brassica* whole-genome triplication event is estimated to have taken place approximately 9–15 Mya [14,34]. Overall, these results indicate the divergence of the *Brassica* genus from Arabidopsis, followed by whole-genome triplication, and hybridisation of *B. rapa* and *B. oleracea* to form *B. napus*, as well as gene loss and rearrangements that shaped the *Dof* gene family in *B. napus*.

### 2.6. Functional Annotation of BnaDofs and Promoter Analysis

Functional annotation allows detailed evaluation of proteins with unidentified molecular function, biological processes, or cellular components. In the cellular component gene ontology category, all the *BnaDof*s were associated with “nucleus”, and the majority of them were associated with “integral component of membrane” terms. A *Dof* gene family is a transcription factor family. It was expected that in the molecular process category, the *BnaDof*s would be associated with terms such as “DNA binding” and “DNA-binding transcriptional factor activity”. In the biological process category, *BnaDof*s were associated with “regulation of transcription” and several other terms related to organ development, vegetative to reproductive transition, light signalling, response to different hormones, cell differentiation, and oxidation–reduction, among others. A detailed summary of functional annotation results along with descriptors is provided in Table S7.

**Figure 6:**
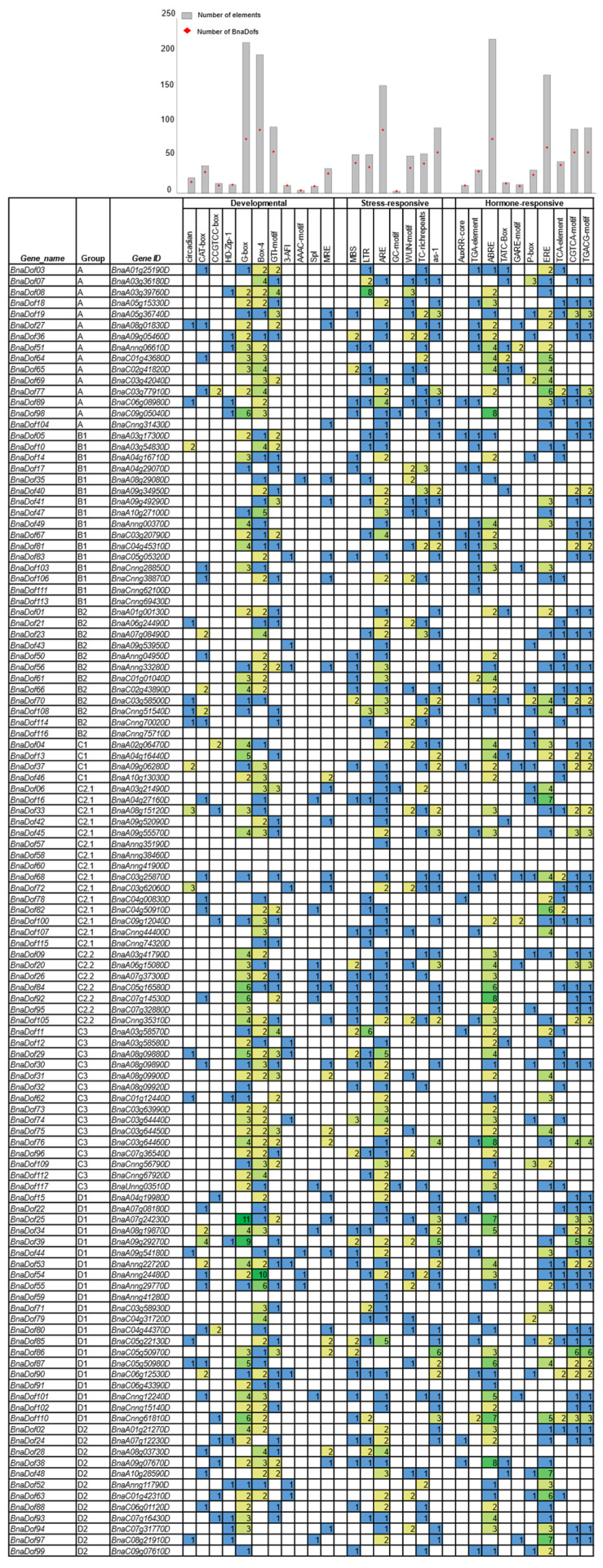
Cis-acting regulatory elements in the promoter region of *BnaDof* genes. The cis-acting regulatory element analysis in the promoter region (1.5 kb upstream of translation initiation site) of *BnaDof* genes was performed using the PlantCARE database. The number of each cis-acting element in the promoter regions of *BnaDof* genes are represented in three major categories: developmental, stress-responsive, and hormone-responsive. On top, the bar graph represents the total number of each cis-acting element present in *BnaDof*s (grey box) and the corresponding number of *BnaDof*s promoters carrying a particular cis-element (red diamond). The details of the cis-elements are provided in Table S10.

To gain further understanding of the functional roles of *BnaDof*s, we used the PlantCARE database to identify potential cis-regulatory elements present upstream of the coding regions (1.5 kb upstream) [35]. Several cis-acting regulatory elements were found in the promoter region of *BnaDof*s*,* and we classified them into three categories: developmental, stress-responsive, and hormone-responsive (Figure 6). Among the development-related cis-elements, we identified elements regulating light responsiveness (G-box, Box-4, GT1-motif, 3-AF1, AAAC-motif, Sp1, and MRE), circadian rhythm (circadian), meristem expression (CAT-box) and differentiation of palisade mesophyll cells (HD-Zip-1). Cis-acting regulatory elements related to light responsiveness, especially the G-box, Box-4, and GT1-motif elements, were present in ~66%, ~77%, and ~50% *BnaDof*s. CCGTCC-box, a development-related cis-element, was also found in the promoters of 11 *BnaDof*s, out of which seven *BnaDof*s belonged to the D major group.

Furthermore, we detected the presence of the stress-responsive cis-elements MBS (involved in drought inducibility), LTR (low-temperature responsive), WUN-motif (wound responsive), TC-rich repeats (defence and stress-related), ARE (anaerobic induction), and GC-motif (anoxic specific inducibility) in 43, 37, 36, 42, 90, and 3 *BnaDof* promoters, respectively. An as-1 cis-element reported to be present in pathogenesis-related genes in plants was also detected in the promoters of 58 *BnaDof*s. Stress signalling and hormone signalling operate at an intertwined level in the regulation of plant stress-responsive gene expression. Therefore, we identified hormone-responsive cis-elements in the *BnaDof* promoters. Among all the hormone-responsive elements, 219 ABRE (Abscisic acid Responsive Elements) elements were present in 77 (68%) *BnaDof*s. Cis-elements responsive to auxin (AuxRR-core and TGA-element), gibberellin (TATC-box, GARE-motif, and P-box), salicylic acid (TCA-element), ethylene (ERE), and methyl jasmonate (CGTCA and TGACG) were also present in the *BnaDof* promoters. In addition to the above-mentioned cis-elements, we encountered several other cis-acting elements such as the AE-box (part of a module for light response), MYB, Myb-binding site, MYC, TATA-box, and CAAT box, which was present in the *BnaDof* promoters.

### 2.7. Tissue-Specific and Abiotic Stress-Responsive Expression Profiling of BnaDofs

**Figure 7:**
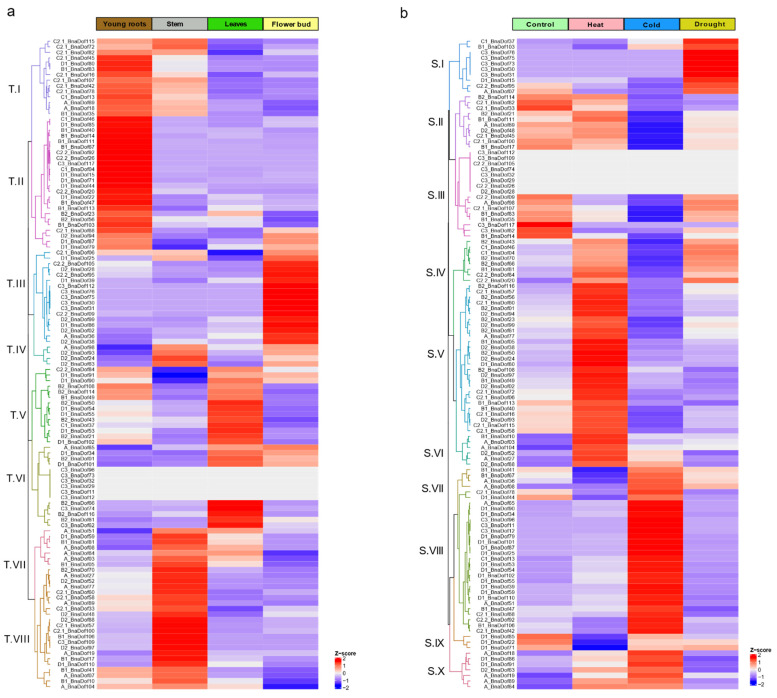
(**a**) Heat map represents the tissue-specific expression of *BnaDof*s. RNA-Seq data were obtained from root, stem, leaf, and flower of *B. napus*. (**b**) Heat map representation of the stress-responsive expression of *BnaDof*s. RNA-Seq data were obtained from three weeks old *B. napus* seedlings grown at control conditions (16/8 h photoperiods at 22 °C, RH 50%, and a light intensity of 230–240 μmol m^−2^ s^−1^) and exposed to Heat: 35 °C for 24 h, Cold: 4 °C for 24 h, and Drought: 22 °C, 25% PEG-6000 for 24 h. Hierarchical clustering of *BnaDof* expression profiles was performed using the Euclidean distance method and complete clustering method. The scale bar represents the Z-score (scaled TPM values).

To analyse the tissue-specific expression patterns of *BnaDof*s, publicly available RNA-Seq data [14] for four tissues were compared: young root, stem, leaf, and flower buds (Table S10a). The distinctive tissue-specific expression of *BnaDof*s could be grouped into eight (T.I–T.VIII) clusters, as illustrated in Figure 7a. The majority of the *BnaDof*s belonging to cluster T.II showed higher expression in young roots. Similarly, T.III and T.VIII showed higher expression in flower buds and stem, respectively. Additionally, *BnaDof*s belonging to cluster T.I, T.IV, T.V, and T.VII showed higher expression in at least two tissues. Interestingly, in cluster T.VI, *BnaDof01, BnaDof34, BnaDof65,* and *BnaDof101* had higher expression in leaves and flower buds, *BnaDof61, BnaDof62, BnaDof66, BnaDof74,* and *BnaDof116* showed higher expression in leaves, whereas the expression of *BnaDof11, BnaDof12, BnaDof29, BnaDof32, BnaDof73,* and *BnaDof96* was undetectable in any of the four tissues. Preferential expression within the tissue-specific cohort of the *Dof* family group was also noticeable. For instance, *BnaDof* members belonging to group C2.1 showed higher expression in young roots and stem; more than 50% of the A and B1 *Dof* group members showed preferential expression in stem and young roots, respectively. 

Brassica Expression DataBase (BrassicaEDB), a gene expression database for Brassica crops (version 1.0) was released recently [36]. To get a comprehensive understanding of developmental expression patterns of *BnaDof*s, we compiled the expression maps of *BnaDof*s, across different tissues and developmental stages as available on Brassica EDB (Supplementary Folder). The expression levels of *BnaDof11, BnaDof12, BnaDof29, BnaDof32, BnaDof73,* and *BnaDof96* were undetectable in young roots, stem, leaves, and flower buds based on our RNA-Seq analysis (Figure 7a). Thus, we observed the expression maps of these genes on BrassicaEDB. *BnaDof73* showed limited expression in cotyledons (48 h after germination), seeds (13 days after fertilisation), and the seed coat (inner integument). Similarly, *BnaDof96* and *BnaDof11* were only expressed in the seed coat (inner integument), *BnaDof32* and *BnaDof29* in seeds 13 days after fertilisation, and *BnaDof12* in seeds 10 days after fertilisation.

We further investigated the changes in expression patterns of *BnaDof*s in three-week-old *B. napus* seedlings exposed to various abiotic stresses by analysing the publicly available RNA-Seq data [36]. Figure 7b illustrates the expression patterns of *BnaDof*s in control conditions and upon exposure to heat stress, cold stress, and drought conditions (Table S10b). The expression profiles of *BnaDof*s were grouped into ten clusters (S.1–S.X). Numerous *BnaDof* genes showed changes in expression profiles upon exposure to extreme temperature. *BnaDof*s grouped in clusters S.VI, S.VII, S.VIII, and S.X were upregulated in response to low-temperature stress. *BnaDof* genes belonging to clusters SII, SIII, SIV, SV, and S.IX were downregulated in response to cold stress. The expression of some *BnaDof*s belonging to cluster S.III (*BnaDof 26, BnaDof28, BnaDof29, BnaDof32, BnaDof74, BnaDof105, BnaDof109*, and *BnaDof112*) was undetectable or did not show any changes in expression upon abiotic stress exposure. This probably indicates that these genes might not be involved in heat, cold, and drought stress response in three-week-old seedlings. Cluster S.IV, S.V, and S.VI genes were upregulated upon exposure to heat stress. The majority of the heat-responsive *BnaDof*s were upregulated. Interestingly a fraction of *BnaDof*s*,* which were upregulated in response to heat stress, showed downregulation in response to cold stress and vice versa. For example, *BnaDof*s clustered in S.IV were upregulated in response to heat and downregulated in response to cold, and an opposite trend was seen in genes clustering in S.VII. Genes belonging to clusters S.I and S.IV showed significant upregulation under drought conditions, and S.II cluster *BnaDof*s were downregulated. However, the majority of *BnaDof*s were not drought-responsive. 

## 3. Discussion

*Brassica napus*, the second-largest economically important oilseed crop, is an allopolyploid formed due to the spontaneous pairwise hybridisation of *B. rapa* and *B. oleracea*. The availability of the *B. napus* genome provides opportunities for the identification of important gene families. One of the important plant-specific transcription factor families is the *Dof* zinc finger gene family. This gene family has been described in several plant species, including Arabidopsis [6], rice [7], wheat [8], tomato [37], pepper [38], Chinese cabbage [21], and cucumber [39], among others, but not in *B. napus*. Functional characterisation of various DOF proteins has highlighted their role in key plant functions such as seed development and germination [40,41], light-regulated hypocotyl elongation [42], photosynthesis [10,43], flowering [44,45,46], lipid biosynthesis [47,48], carbon metabolism [5], pollen development [49], abiotic stress response [46,50,51], and several other biological processes. In this study, we performed in silico genome-wide identification, comparison, and evolutionary analysis of the *Dof* gene family in *B. napus*. Here, we report 117 genes as putative members of the *Dof* gene family in *B. napus,* which is the largest number of *Dof* genes ever reported in eudicots.

### 3.1. Systematic Analysis of BnaDofs

According to the phylogenetic analysis of *Dof* genes in Arabidopsis and rice, reported by Lijavetzky and collaborators [7], members of the *Dof* gene family are classified into nine groups. The 117 *BnaDof*s identified in our study were also classified into nine groups based on the phylogenetic analysis between the identified *BnaDof*s and Arabidopsis *Dof* genes. We further performed a phylogenetic analysis between Arabidopsis, *B. napus*, *B. rapa,* and *B. oleracea*. Our present study also identified 62 *Dof* genes in *B. oleracea*. In *B. rapa*, 76 *Dof* genes were reported [21]; however, we included only 74 *B. rapa Dof* in our analysis. *Bra007632* contained a B3 DNA binding domain, auxin response factor domain, Aux/IAA domain, and DOF domain and was orthologous to *AUXIN RESPONSE FACTOR 18* Arabidopsis. *Bra002057* contained a Syntaxin domain and the DOF domain and was orthologous to a *SYNTAXIN OF PLANT 21* gene in Arabidopsis. Thus, these two genes were excluded from our study. The *Dof* gene family across the *Brassica* species also clustered phylogenetically in nine groups. Furthermore, comparative analysis of the gene structure and conserved protein domains highlighted conserved exon-intron organisations and distribution of protein motifs followed by most BnaDofs. The DOF domain (motif 1, Figure 3c) was conserved across all BnaDofs, and a similar motif distribution pattern can be seen across the members belonging to the same group. The presence of specific motifs across members of a subgroup, for example, motif 7 being only present in the members of subgroup C3, indicates the specificity of these motifs to the evolution of a subgroup. 

The exon-intron organisation of most *BnaDof*s was similar to *Dof* genes reported in other plants such as Arabidopsis and rice [7]. The majority of the *BnaDof* genes had zero to one intron. Eight *BnaDof*s had two introns, and two *BnaDof*s (*BnaDof40* and *BnaDof86*) had three introns. The intron length across members of a subgroup was also diverse. In Arabidopsis, the *Dof* genes had zero to one intron, and in rice, the *Dof* genes had zero to two introns [7]. It is worth mentioning that in Arabidopsis, the transcripts/splice variants of *Dof* genes can have more than one intron; for example, *At3g55370.3*, a transcript of *At3g55370,* had three introns. We also observed the exon-intron organisation of *B. rapa* and *B. oleracea Dof* genes (Figure S2). *B. rapa Dof* genes had zero to two introns and *B. oleracea Dof* genes had zero to one intron. The structural diversity of *BnaDof* genes in terms of intron number suggests that in comparison to the *Dof* genes of *B. rapa* and *B. oleracea*, *BnaDof*s acquired introns during evolution. Li et al. 2019 [52] reported that the EIN/EIL3 gene family members in *B. napus* also acquired introns. The presence of introns can be advantageous for an organism [53]. For instance, due to alternative splicing, the protein diversity of an organism can increase. Introns are also reported to regulate gene expression and produce non-coding RNAs that play diverse regulatory roles.

### 3.2. Expansion and Divergence of the Dof Gene Family in B. napus

Genome-wide studies identifying gene families in *B. napus* have reported the frequent expansion of gene families such as HSF, GST, CRF, TLP, and bHLH, to name a few [54,55,56,57,58]. The difference in the size of the *Dof* gene family from Arabidopsis to *B. napus* indicates the expansion of the *B. napus Dof* gene family. We performed orthologous gene clustering, synteny analysis and molecular evolutionary analysis to understand the expansion of the BnaDofs. The average synonymous base substitution rate between *B. napus Dof* genes and their Arabidopsis orthologs was calculated as 0.51 (0.28 to 0.74). We further estimated the divergence time of ~17 Mya, and it was constant with the time Arabidopsis and *Brassica* lineages diverged, i.e., 14–24 Mya [33]. Similarly, the calculated maxima, minima, and average Ks values of the orthologous gene pairs between the *Brassica* species highlights that the whole-genome triplication events (9–15 Mya) and the hybridisation event (7500 years ago) led to the expansion of the *Dof* gene family in *B. napus* [14,32]. The expansion of gene families due to whole-genome and local gene duplication events might be an effective strategy in plants for adapting to the ever-changing environmental conditions.

### 3.3. Distinct Expression Patterns of BnaDofs during Development

Tempo-spatial expression profiles of *BnaDof*s in association with functional annotations and cis-element analysis of *BnaDof* promoters indicate their preferential expression in different tissues, suggesting a diversification of function during organ development. The functional characterisation of DOF proteins in different plants has indicated their association with light-responsiveness, phytochrome signalling, seed germination, and tissue-specific expression in endosperms, vascular tissue development, leaves, or guard cells [11,59,60,61]. Here in this section, to provide better clarity, we will be discussing the *BnaDof* genes in terms of orthologous relationships with Arabidopsis *Dof* genes rather than in terms of classified groups, as different schemes for the *Dof* gene family classification are available in the literature [6,7,11]. 

Light is an indispensable environmental cue regulating developmental processes such as photomorphogenesis, seed germination, flowering, and several other metabolic and cellular processes [62]. In plants, the first DOF TF reported in maize was involved in light signalling [3,4,5]. The *BnaDof* promoters are enriched for the presence of light-responsive cis-acting elements. Several *Dof* genes in plants are associated with the regulation of photomorphogenesis, seed germination, and development [59,60]. 

Light is essential for converting the inactive Pr form of phytochrome into the active Pfr, which then activates the process of seed germination [63]. In Arabidopsis, *DAG1, DAG2, COG1,* and *OBP3* were reported to regulate seed germination and hypocotyl elongation [64,65,66,67]. *DAG1* represses seed germination in response to light, and *DAG2* activates seed germination, thus acting antagonistically [64]. In Arabidopsis, the expression of *DAG1* and *DAG2* is detected in vascular tissues but not in seeds, suggesting regulation of long-distance light-related signalling pathways. The *B. napus* gene orthologous to *DAG1* is *BnaDof45,* and to *DAG2* are *BnaDof06* and *BnaDof68*. These genes also showed comparatively higher expression in tissues other than seed or embryo. 

The first reported DOF protein to regulate phytochrome-mediated signalling involved in seedling development was *COG1* [65]. *COG1* interacts with *Phytochrome Interacting Factors* (*PIF4* and *PIF5*), activates Brassinosteroids biosynthesis, and promotes hypocotyl elongation [68]. It was also reported that *COG1* controls the expression of *PRX2* and *PRX25,* which are associated with seed longevity, and thereby regulates seed tolerance [69]. Four *BnaDof* genes, *BnaDof22, BnaDof44, BnaDof71,* and *BnaDof85,* were identified as orthologous to *COG1*(*At1g29160*) and functionally annotated to be associated with seed coat development, and showed very high to moderate expression in the seed coat. The Arabidopsis *OBP3* gene is involved in hypocotyl elongation repression in a light-dependent manner [67]. Among the orthologs of *OBP3*, in comparison to *BnaDof111*, *BnaDof40* showed higher expression in hypocotyl, suggesting a similar function. 

Circadian rhythms occur in plants to respond to daily and seasonal changes and synchronise their developmental programme based on the day length [70]. In addition to light-responsive cis-elements, a few *BnaDof* promoters also showed the presence of circadian clock associated cis-elements (Figure 6). In Arabidopsis, cycling DOF factors (*CDF1, CDF2, CDF3,* and *CDF5*) were reported to regulate the photoperiodic flowering response [44,71]. In Arabidopsis, *CDF1* represses the transcription of a core circadian clock signalling gene *CONSTANS* (*CO*) [44,72]. The *CO* gene is involved in regulating flowering under long days, and its repression by *CDF1* represses flowering in Arabidopsis [44,73]. Its ortholog in *B. napus*, *BnCDF1* (*BnaDof54* in our study), was also reported to play a role in flowering [74]. *BnaDof54*, functionally annotated to be involved in flower development, shows very high expression in flowers. Similarly, *BnaDof55,* which is also orthologous to *CDF1*, was functionally annotated to be related to flower development and showed higher flower expression, indicating a similar functional role.

The Arabidopsis *Dof* gene, *At3g45610*, also known as *Dof6* or *Dof3.2*, negatively regulates seed germination [75]. Orthology analysis revealed the absence of the *Dof6* orthologous gene across the *B. napus Dof* gene family. Gene loss due to the evolution and divergence of *BnaDof* genes might have either resulted in a loss of function or neofunctionalisation. It is worth mentioning that the cis-element analysis of the *BnaDof* promoters also revealed cis-elements related to or acting as binding sites for other transcription factors (MYB, MYC, ARF, and MADS-boxes). A similar diversification of binding sites was reported for Arabidopsis *Dof* promoters, indicating potential relationships between DOF TFs and other TFs regulating diverse plant developmental processes [76].

In cereals, *Dof* genes play a role in seed protein accumulation and mobilisation [60,77,78]. In maize, *ZmDof36* and *ZmDof3* play a role in seed starch accumulation [79,80]. However, for *B. napus,* the accumulation of oil in the seeds is of economic importance. In soybean, two DOF-like proteins, GmDOF4 and GmDOF11, enhance the fatty acid content in seeds when overexpressed in Arabidopsis by directly binding to the promoter regions of the acetyl CoA carboxylase gene and long-chain-acyl CoA synthetase gene and activating their expression [47]. Similarly, the overexpression of the *GhDof1* gene in cotton can potentially increase seed lipid content [48]. Thus, further exploring the role of *BnaDof* genes in regulating seed oil content will prove beneficial in producing high oil yielding varieties.

### 3.4. Potential Role of BnaDofs in Abiotic Stress Response

DOF TFs have also been reported to participate in response to the abiotic stress response [62]. Genome-wide expression analysis studies have reported the abiotic stress-responsive gene expression of *Dof* genes in Chinese cabbage [21], wheat [8], tomato [45], pepper [38], rose [81], and other plant species. We investigated the expression profiles of *BnaDof*s in response to heat, cold, and drought stress. In response to heat stress, the majority of the differentially regulated *BnaDof*s were upregulated. The mechanism by which *Dof* genes regulate heat stress response is not yet described for *Dof* genes. In walnut, Yang et al. [82] suggested the contribution of *JrDof3* in enhancing the heat stress response of *JrGRAS2*. *JrGRAS2* overexpression lines in Arabidopsis exhibited enhanced heat stress tolerance.

The majority of *BnaDofs* in our analysis were differentially regulated upon exposure to low temperatures. A recent study in *B. napus* exploring the cold-responsive TFs reported the changes in expression of *Dof* genes in response to the cold stress response and suggested their possible role in imparting cold tolerance [50]. In cotton, the overexpression of the *GhDof1* gene enhanced cold tolerance during the seedling stage [48]. The transgenic line overexpressing *GhDof1* also exhibited enhanced salinity tolerance due to enhanced root development in transgenics under salt stress. 

In *B. napus*, *BnCDF1* (*BnaDof54* in our study) has been reported to play a role in freezing tolerance [74]. Based on our expression analysis, this gene was highly upregulated upon exposure to 4 °C for 24 h. Furthermore, the overexpression of two tomato *CDF* genes (*SlCDF1* and *SlCDF3*) in Arabidopsis enhanced drought and salt tolerance [45]. The overexpression of Arabidopsis *CDF3* also enhanced the tolerance of transgenic Arabidopsis plants to drought, cold, and osmotic stress [46]. A tomato transgenic line overexpressing *AtCDF3* and *SlCDF3* exhibited an enhanced growth rate and yield under control and salt stress conditions [83]. Transcriptomic analysis of these transgenic tomato lines revealed the role of *CDF3* in regulating the expression of several genes involved in cell growth, metabolism, and stress response. The *CDF3* orthologous genes in *B. napus*, *BnaDof53* and *BnaDof102,* significantly upregulated in response to cold stress and slightly in response to heat stress. In comparison to control conditions, these two genes showed a slight reduction in gene expression under drought. *CDF*s thus play a potential role in abiotic stress tolerance, in addition to their role in flowering time control. 

We further observed a few *BnaDofs* (−17, −47, −81, and −113) associated with the functional terms “oxidation–reduction process” and “response to oxidative stress”, suggesting a potential role in ROS-mediated signalling. In wheat, some *TaDof*s have been suggested to act as dynamic regulators of ROS clearance pathways based on their response to heavy metal stress [8]. DOF proteins are also involved in phytohormone signalling pathways [84]. Our cis-acting element analysis of the *BnaDof* promoters and the functional annotation of BnaDof proteins revealed the presence of several cis-elements responsive to auxin, abscisic acid, salicylic acid, gibberellic acid, and methyl jasmonate. ABRE elements were enriched in the promoters of 77 *BnaDof*s. ABA-dependent pathways and phytohormone signalling are known to respond to abiotic stresses [85,86]. This suggests that hormones can activate *BnaDof* expression, and they might play a role in stress signalling pathways. 

The expression analysis exemplifies the stress-responsive nature of *BnaDof*s. The differential regulation of *BnaDof*s may regulate downstream genes involved in stress response, probably imparting tolerance. Different *BnaDof*s can be stress-responsive in different tissues and at different developmental stages, due to the preferential tissue expression of *BnaDof*s. It is also important to note that depending upon the variety and even plant species, the expression profiles and functional roles of *Dof* genes in response to stress may show variation. Overall, this study provides a comprehensive understanding of the molecular structure, evolution, and potential functions of *BnaDof*s.

## 4. Materials and Methods

### 4.1. Identification of Dof Gene Family Members in B. napus

A BLASTP search of the *B. napus* proteome was carried out using zf-DOF-domain search model accession (Pfam: PF02701) as a query to obtain the consensus amino-acid sequences of the putative DOF proteins. The term ‘Dof’ and DOF-domain search model accession ‘PF02701′ were used to search the Plant Transcription Factor Database 4.0 database (PlantTFDB) [18]. To identify the integrated DOF domain in the putative DOFs obtained from the BLASTP and PlantTFDb search, SMART 8.0 software (http://smart.embl-heidelberg.de/, accessed on 5 August 2020) was used, and the final predicted DOFs were further characterised [19]. Expasy server’s ProtParam tool (https://web.expasy.org/protparam/, accessed on 7 August 2020) was used to compute the various physical and chemical properties of the predicted Dof proteins such as the number of amino acids in the protein sequence, molecular weight (Mw), protein isoelectric point (pI), and Grand Average of Hydropathy (GRAVY) of the protein [87]. Chromosomal locations as well as the genomic, coding, peptide, and promoter sequences of the DOF TFs were downloaded from GenoScope (*Brassica napus*. annotation_v5) database. Unique gene identifiers were assigned to the DOFs, and they were referred to as BnaDofs.

### 4.2. Evolutionary and Gene Duplication Analysis of BnaDofs

Multiple sequence alignments were performed on the DOF amino acid sequences using CLUSTALW with default settings [23]. MEGA7.0 was used to construct a phylogenetic tree based on the maximum likelihood method based on the JTT matrix-based method. Statistical support for each tree node was provided by performing a 100–500 replicate bootstrap analysis [24]. We also constructed the phylogenetic tree of BnaDofs using the neighbour-joining (NJ) method with 1000 bootstrap replicates, and the Poisson correction method.

To identify gene duplications in *BnaDofs*, all *B. napus* gene sequences (101040) were first aligned using BLASTp, with an e-value of 1e-10, and then the duplication patterns were classified into interspersed and tandem duplications with MCScanX (default parameters) [20,27]. Gene duplication was also analysed based on sequence similarity criteria, i.e., the similarity of the aligned regions of protein ≥80% [40]. Evolutionary analyses were conducted in MEGA7 [24]. The number of synonymous substitutions per synonymous site (dS/Ks), and the number of non-synonymous substitutions per non-synonymous site (dN/Ka), were calculated using the Nei–Gojobori method (Jukes–Cantor). The formula T  =  Ks/2R (where, Ks = number of synonymous substitutions per synonymous site, R = 1.5 × 10^−8^ synonymous substitutions per site per year, and T = divergence time) was used to estimate divergence time [33,88]. 

### 4.3. Gene Structure and Motif Analysis of BnaDofs

The gene structure in terms of the exon-intron organisation was determined using the GSDS2.0 (Gene Structure Display Server; http://gsds.cbi.pku.edu.cn, accessed on 23 September 2020) [73,89]. The MEME tool from the MEME suite 5.1.1 (http://meme-suite.org/tools/meme) was used to identify fifteen statistically significant motifs of the BnaDof protein sequences based on “zero or one occurrence per sequence (zoops)” [25]. The discoverable motif length and sites were set to 6–50 and 2–600, respectively.

### 4.4. Functional Annotation and Promoter Analysis

Functional annotation of BnaDofs was performed using PANNZER2 (Protein ANNotation with Z-scoRE 2), which provided both Gene Ontology (GO) annotations and free text description predictions [90]. Promoter analysis of the *BnaDof* genes was performed by using the PlantCARE database (http://bioinformatics.psb.ugent.be/webtools/plantcare/html/, accessed on 25 September 2020) to identify the cis-acting regulatory elements with putative involvement in various abiotic stress responses [35]. 1500 bp upstream regions from the start codon (ATG) of the *Dof* genes were downloaded as the promoter sequences from the EnsemblPlants Database [91]. 

### 4.5. Orthology and Collinearity Analysis of BnaDofs

Genome annotations and peptide sequences were downloaded from GenoScope (*Brassica_napus*.annotation_v5) and EnsemblPlants (*Brassica_rapa*.IVFCAASv1.36, *Brassica_oleracea*.v2.1.36). The orthologous genes in *B. oleracea*, *B. rapa*, *B. napus,* and *A. thaliana* were identified using OrthoVenn2 (https://orthovenn2.bioinfotoolkits.net/home, accessed on 27 September 2020) [26]. 

For the analysis of collinearity/synteny, 58 out of 117 DOF transcription factor members identified in *B. napus* were discarded because their chromosome of origin was uncertain. Syntenic blocks were identified by a sequence similarity search of the remaining 59 DOF members in *B. napus* against the reference genomes of Arabidopsis, *B. rapa,* and *B. oleracea*, using Blastn v. 2.10.1+ with stringent parameters (cut-off e-value 10e-50nd a minimum percentage of identity of 75) [20]. A custom python script was used to reformat the blast output and identify collinear genes and genes that underwent chromosomal translocation. Synteny plots were plotted with CIRCOS v. 0.69–9 [92].

### 4.6. Tissue-Specific Expression and Abiotic Stress Response Expression Profiling of BnaDofs

To investigate tissue-specific expression (under non-stressed conditions) and abiotic stress-responsive expression of the *BnaDof* genes, RNA-Seq data sets from previously published literature [14,36] were downloaded from the NCBI Sequence Read Archive database (Table S11). Transcript expression was quantified using Kallisto v0.44.0, and read abundance was expressed as Transcripts Per Kilobase Million (TPM) [93]. Heat maps were drawn by using the ComplexHeatmap package to visualise the expression of *BnaDof*s [94]. Additionally, the expression maps for *BnaDofs* were downloaded from the Brassica expression DataBase (BrassicaEDB). The Supplementary Folder with the downloaded expression maps of *BnaDof*s is accessible via the following link https://jmp.sh/odBHlfT.

## 5. Conclusions

A systematic analysis of the *B. napus Dof* transcription factor gene family identified a total of 117 *BnaDof*s. The *BnaDof*s were classified into nine groups: A, B1, B2, C1, C2.1, C2.2, C3, D1, and D2 based on the phylogenetic analysis. Based on the orthology, synteny, and evolutionary analysis, the calculated divergence times indicated that the divergence of the *Brassica* and Arabidopsis genus (~17 Mya), the whole-genome triplication event (9–15 Mya), and the formation of *B. napus* (7500 years ago) drove the expansion of the *BnaDof* gene family. Synteny analysis also highlighted that the majority of the *Dof* genes with known chromosomal locations in *B. napus* did not undergo translocations. The Ka/Ks ratio of the duplicated gene pairs indicated that the *BnaDof* gene pairs underwent purifying selection. Further understanding of the molecular evolutionary mechanism is required to understand how gene duplications, gene loss, and rearrangements can lead to the expansion of gene families and the possible neo- or sub- functionalisation of genes. Tissue-specific expression highlighted the role of *BnaDof*s in organ development and other developmental processes. Most of the *BnaDof*s were responsive to temperature fluctuations and were differentially regulated, particularly by cold stress. Additionally, molecular characterisation, functional annotation, and cis-acting element analysis have provided a starting point for further research investigations—our study supports the involvement of the *Dof* gene family in developmental processes and multiple abiotic stress responses. Further research is warranted to dissect the role of *BnaDof*s and explore these transcriptional regulators for developing climate change-resilient varieties with desirable physiological and agronomic traits.

## Data Availability

All data generated or analysed during this study are included in this article and its supplementary information files. Details and accession numbers of the RNA-Seq data libraries downloaded from NCBI sequence Read Archive are outlined in Table S9. The expression maps of *BnaDof*s downloaded from the Brassica Expression DataBase (BrassicaEDB) are accessible via the following link https://jmp.sh/odBHlfT.

## Supplementary Materials

The following are available online at https://www.mdpi.com/article/10.3390/plants10040709/s1: Figure S1. Phylogenetic tree of Arabidopsis, *B. napus, B. oleracea,* and *B. rapa* DOF proteins. The evolutionary history was inferred by using the maximum likelihood method based on the JTT matrix-based method. Maximum-likelihood bootstrap values (100 replicates) above 70% are shown. The analysis involved 289 amino acid sequences. There was a total of 651 positions in the final dataset. Phylogenetic topology was generated via MEGA7. Figure S2. Gene structure of (a) *B. rapa Dof* genes, (b) *B. oleracea Dof* genes. Table S1. Information of BnaDofs, including gene ID, type of *Dof*, chromosomal location, protein sequence length, isoelectric point (pI), molecular weight (Mw), instability index (I.I), stability, aliphatic index (A.I), and grand average of hydropathicity (GRAVY). Table S2. Summary of *Dof* genes identified in *B. oleracea* and reported *Dof* genes in *B. rapa* and Arabidopsis. Table S3. Detailed summary of identified syntenic genes between A sub-genome of *B. napus* and *B. rapa*, and C sub-genome of *B. napus* and *B. oleracea*. Table S4a. Detailed list of orthologous gene clusters between the *Dof* genes in *B. rapa*, *B. oleracea*, *B. napus,* and Arabidopsis. Table S4b. Detailed list of singleton *Dof* genes in Arabidopsis, *B. napus,* and *B. rapa*. Table S5a. Detailed summary of gene duplications in *BnaDofs* based on similarity criteria (≥80%). Table S5b. Detailed summary of gene duplications along with type of duplications in *BnaDof*s as predicted by MCScanX. Table S6a. Orthologous gene pairs of Arabidopsis and *B. napus Dof* genes and their respective Ks values. Table S6b. Orthologous gene pairs of *Dof* genes across the three *Brassica* species (*B. napus*, *B. oleracea,* and *B. rapa*) and their respective Ks values. Table S7. Detailed results of functional annotation of BnaDof proteins based on PANNZER2 output. Table S8. Cis-acting regulatory elements detected in the *BnaDof* promoters. Table S9. Description of cis-acting regulatory elements detected in the *BnaDof* promoters (used in Figure 6). Table S10a. Tissue-specific RNA-Seq expression data. Read abundance is showed in terms of Transcripts Per Kilobase Million (TPM). Table S10b. RNA-Seq expression data for three-week-old *B. napus* seedlings grown in control conditions and exposed to different abiotic stresses. Read abundance is showed in terms of Transcripts Per Kilobase Million (TPM). Table S11. Summary of the sequencing data used for analysis. Supplementary Folder: Expression maps of *BnaDofs* (https://jmp.sh/odBHlfT).
